# Vitellogenin 2 promotes muscle development and stimulates the browning of white fat

**DOI:** 10.18632/aging.203590

**Published:** 2021-10-05

**Authors:** Yilei Li, Xiaoli Sun, Yun Bai, Yunyan Ji, Huawei Ren, Xiuju Yu, Yi Yan, Xiaoyan He, Yanjun Dong, Liping Zhang, Xiaomao Luo, Haidong Wang

**Affiliations:** 1 College of Veterinary Medicine, Shanxi Agricultural University, Taigu, Shanxi 030801, China; 2 Department of Respiratory and Critical Care Medicine, Shanghai Pulmonary Hospital, Tongji University School of Medicine, Shanghai 200433, China; 3 College of Veterinary Medicine, China Agricultural University, Beijing 100193, China; 4 Nephrology Division, Department of Medicine, Baylor College of Medicine, Houston, TX 77030, USA

**Keywords:** browning, skeletal muscle, FEYE, proliferation and differentiation, VTG2

## Abstract

Eggs are rich in nutrients and contain a lot of protein. Although eggs have proved to accelerate the growth of C2C12 cells, the regulatory and mechanism of fertilized egg yolk extract (FEYE) on skeletal muscle development and fat metabolism remains unclearly. The mice were treated with FEYE by gavage for 24 d, we found that FEYE can inhibit the expression of skeletal muscle atrophy genes such as MSTN and Murf-1, and up-regulate the expression levels of MYOD, MYOG and Irisin. In addition, the treatment of FEYE induced UCP1 and PGC1α high expression in WAT, thereby causing WAT browning reaction. In order to confirm the composition of FEYE, we performed protein full spectrum identification (LC MS/MS) analysis and found the most enriched component is vitellogenin 2 (VTG2). Therefore, we added the recombinant protein VTG2 to C2C12 cells and found that VTG2 promoted the proliferation and differentiation of C2C12 cells. After that, we further proved that VTG2 inhibited the expression of MSTN and improved the expression of MYOD and Irisin. Finally, the dual luciferase test proved that VTG2 directly inhibited the transcriptional activity of MSTN. Our results conclude that FEYE inhibits the expression of MSTN in muscle tissues by delivering VTG2, thereby promoting skeletal muscle development, and can also promote the expression level of FNDC5 in serum. Then, FNDC5 acts on the fat through the serum, stimulating the browning reaction of white adipocytes. Therefore, VTG2 can be used to stop muscle consumption, improve skeletal muscle aging, and prevent obesity.

## INTRODUCTION

Skeletal muscle accounts for about 40% of the body weight. The development of animal skeletal muscle determines its economic value [1]. Studies have shown that the number of skeletal muscle fibers is fixed after birth. However, the regulation of certain factors and signals in the growth and development of skeletal muscles can change the regenerative capacity of muscles. MSTN is the main negative regulator of the growth and development of skeletal muscle [2]. Mutation or inactivation of the MSTN gene in cattle, sheep, or human can enhance muscle development [3–6]. On the one hand, MSTN can promote the proliferation of skeletal muscle via activating Smad/Akt signaling pathway [7, 8]. On the other hand, growth differentiation factor 11 (GDF11), insulin-like growth factor-1 (IGF-1), follistatin (FST), G protein-coupled receptor-related sorting protein 1 (GASP-1) and JA16 monoclonal antibodies can inhibit MSTN and indirectly promote muscle development [9–13].

In recent years, the functions of fat have been widely explored. Fat can not only play a role in cancer and immune regulation [14, 15], but also quickly fill wounds and accelerate muscle repair when the body is damaged [16]. Fat tissues in mammals can be divided into WAT and brown adipose tissue (BAT). WAT, which contains many large lipid droplets, is an important endocrine organ for storing energy. BAT contains many small lipid droplets and a large number of mitochondria, which can cause proton leakage in the respiratory chain, thereby releasing heat energy through UCP1 located on the inner mitochondrial membrane. The main function of BAT is to consume energy and produce heat [17–19]. Therefore, the interconversion of different adipose tissues can affect the regulation of the body temperature [20]. BAT primarily exists in the parts of scapular region and WAT [21]. The browning reaction of excess WAT helps to reduce obesity. Interestingly, WAT gradually tends to the characteristics of BAT during the stimulation of exercise, cold, Irisin, BMP7 and other factors [22, 23]. The regulatory effect of signaling pathway on promoting the production of BAT can provide scientific basis for obesity and type II diabetes therapy.

Egg is one of the few global shared foods with high yield and low price. Egg is rich in nutrients, contains a lot of protein, vitamins, and minerals, and has good functional characteristics. The protein metabolism of fertilized egg is very fast. During fertilization, the contents of protein, total amino acids and free amino acids gradually increased and were higher than those in unfertilized eggs [24]. With such high nutrients in eggs, it is unclear whether it can promote muscle development. Vitellogenin (VTG) can produce three subtypes, namely VTG1, VTG2, and VTG3, through different types of alternative splicing. VTG2 has the highest content among the three VTGs. As a precursor of yolk protein, VTG is usually produced in the tissues outside the ovary and gradually transported to the ovary by the circulatory system. VTG is one of the main sources of nutrients for embryonic development [25–27]. VTG was initially believed to be a female specific protein involved in vitellogenesis. However, recent works found that VTG can also be detected in male species under normal physiological conditions, such as in fishes, sea urchins, and crustaceans [28–31]. In addition to participating in the formation of female livetin, VTG may play a role in other organs. Recent research has reported that VTG also plays an important role in innate immune response of different species [32]. However, the role of VTG in muscle development and fat metabolism has been rarely studied.

*In vivo* experiments, we examined the effects of FEYE on body weight, muscle weight, muscle fiber area and gene expression related to muscle proliferation and differentiation in mice. At the same time, we also tested the effect of FEYE on brown fat marker gene expression and mice body temperature. Furthermore, the important regulatory protein in FEYE may be VTG2 by mass spectrometry. Finally, the cells were incubated with VTG2 recombinant protein. In C2C12 cells, our results showed that VTG2 targeted MSTN to promote the proliferation and differentiation of C2C12 cells. In 3T3-L1 cells, we detected that VTG2 could promote the browning reaction of white adipocytes. Our research provides some clues for the regulation mechanism of skeletal muscle development and atrophy, at the same time, we discover new ways of animal body heat production.

## RESULTS

### FEYE promotes the development of mice skeletal muscle

**Figure 1:**
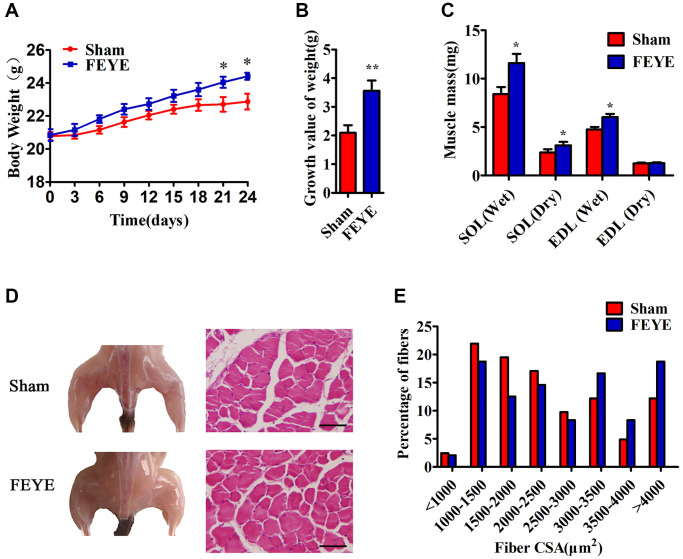
**FEYE promotes muscle growth.** (**A, B**) After the mice are fed with FEYE by gavage for 24 d, compared with Sham group, the body weight (BW) of mice in the FEYE group increases more significantly (**A**), and the weight gain in the FEYE group is higher (**B**) (*n* = 6/group); (**C**) Comparison of dry weight and wet weight of soleus muscle (SOL) and extensor digitorum longus (EDL) in mice between two groups on 24 d (*n* = 6/group); (**D**) FEYE promotes mouse gastrocnemius development; (**E**) Muscle fiber area analysis and muscle fiber size distribution of mice gastrocnemius muscle; (Bar = 100 μm); ^*^P < 0.05, ^**^P < 0.01.

In order to prove the effect of FEYE on the body weight of mice, mice were fed with FEYE by gavage for 24 d. During this period, the mice were given the same forage and housed under the same living environment. The body weight of mice in the FEYE group was significantly higher than that in the sham group (Figure 1A). And compared with sham group, FEYE could obviously increase the growth value of body weight (Figure 1B). In addition, we measured the dry and wet weights of soleus muscle (SOL) and extensor digitorum longus (EDL) by isolating mice muscle. The results showed that the wet weight, dry weight of SOL and the wet weight of EDL in the FEYE group increased significantly (Figure 1C). Compared with the sham group, the area of muscle fiber also increased significantly (Figure 1D, 1E). These results demonstrate that FEYE can increase the weight of skeletal muscle via enlarging muscle fiber area in the mice.

### FEYE inhibits MSTN to promote the proliferation and differentiation of skeletal muscle

**Figure 2:**
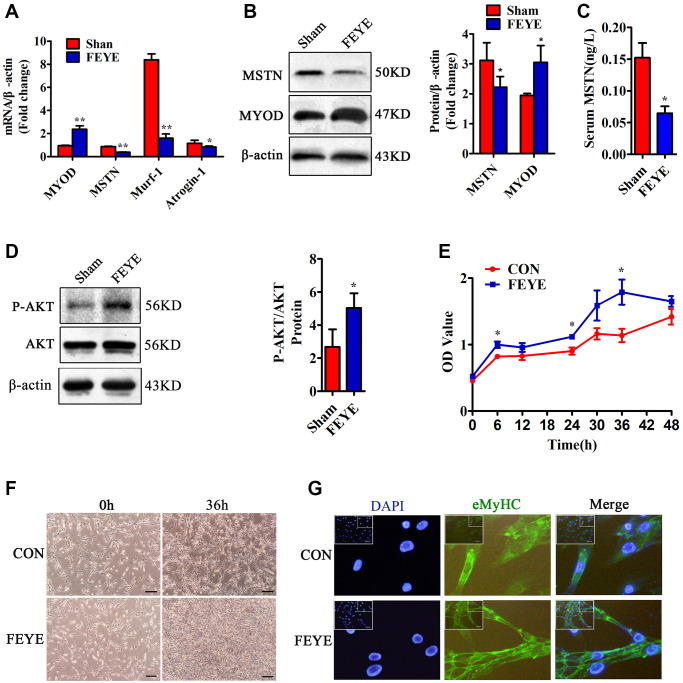
**FEYE promotes the proliferation and differentiation of skeletal muscle.** (**A**) FEYE can effectively inhibit the mRNA expression of MSTN, Murf-1, and Atrogin-1 in skeletal muscle and significantly enhance mRNA expression of myocyte growth factor MYOD. Each experiment was performed in triplicate wells; (**B**) Western blot assay shows that FEYE can significantly inhibit the protein expression of MSTN in skeletal muscle and promote the expression of MYOD. Each experiment was performed in triplicate wells; (**C**) ELISA assay shows that FEYE can decrease the expression level of of MSTN in serum. Each experiment was performed in triplicate wells; (**D**) Western blot assay shows that FEYE can significantly enhance the phosphorylation of AKT. (**E**) CCK-8 assay is used to detect the proliferation rate of C2C12 cells; (**F**) FEYE (10 mg/mL) is added to produce action for 36 h during the proliferation of C2C12 cells. FEYE can promote cell proliferation; (**G**) FEYE is added in the proliferated and differentiated C2C12 cells at the concentration of 10 mg/mL. Immunofluorescence staining of eMyHC shows that FEYE can enhance the formation of myotubes; (Bar = 100 μm); ^*^P < 0.05, ^**^P < 0.01.

Previous study has been proved that MSTN plays a role in negatively regulating muscle development [2]. To prove the influence of FEYE on the expression of muscle-related genes, we detected the expression of MYOD, MSTN, Murf-1 and Atrogin-1 in skeletal muscle tissues. First, the results found that FEYE obviously down-regulated the mRNA levels of Murf-1 and Atrogin-1 in skeletal muscle tissues (Figure 2A). The levels of MSTN mRNA and protein were also suppressed in the FEYE-treated group (Figure 2A, 2B). Meanwhile, the levels of MYOD mRNA and protein in the FEYE-treated group were up-regulated (Figure 2A, 2B). To verify the effect of FEYE on MSTN secretion in the mice serum, the ELISA assay measured the secretion level of MSTN and found that FEYE significantly hindered the secretion of MSTN in the serum (Figure 2C). FEYE not only inhibited the expression of MSTN and other factors, but also activated the PI3K/AKT pathway (Figure 2D). Therefore, FEYE can accelerate the development of skeletal muscle and activate the PI3K/AKT signaling pathway.

In order to prove the effective concentration of FEYE on C2C12 cells, we used the MTT method to find that the proliferation rate of C2C12 increased with the increase of FEYE concentration. Therefore, we determined that the most effective concentration of FEYE is 10 mg/mL (Supplementary Figure 1). Later, it proved the effect of FEYE on C2C12 cells replication *in vitro*. Compared with the control group, we found that FEYE significantly promoted the proliferation of C2C12 cells at 6, 24, and 36 h (Figure 2E). C2C12 cells were treated with FEYE at a concentration of 10 mg/mL for 36 h, and the growth density of cells in the FEYE group was higher than that in the control group (Figure 2F). We incubated C2C12 cells with the same concentration of FEYE and then observed the immunofluorescence staining of eMyHC after cells differentiated. We found that FEYE promoted the formation of C2C12 myotubes (Figure 2G). Therefore, FEYE can promote the proliferation and differentiation of skeletal muscle and myoblasts *in vivo* and *in vitro*.

### FEYE improves the adaptation to cold conditions by increasing the PGC1α and FNDC5 expression which mediate the browning of WAT in mice

**Figure 3:**
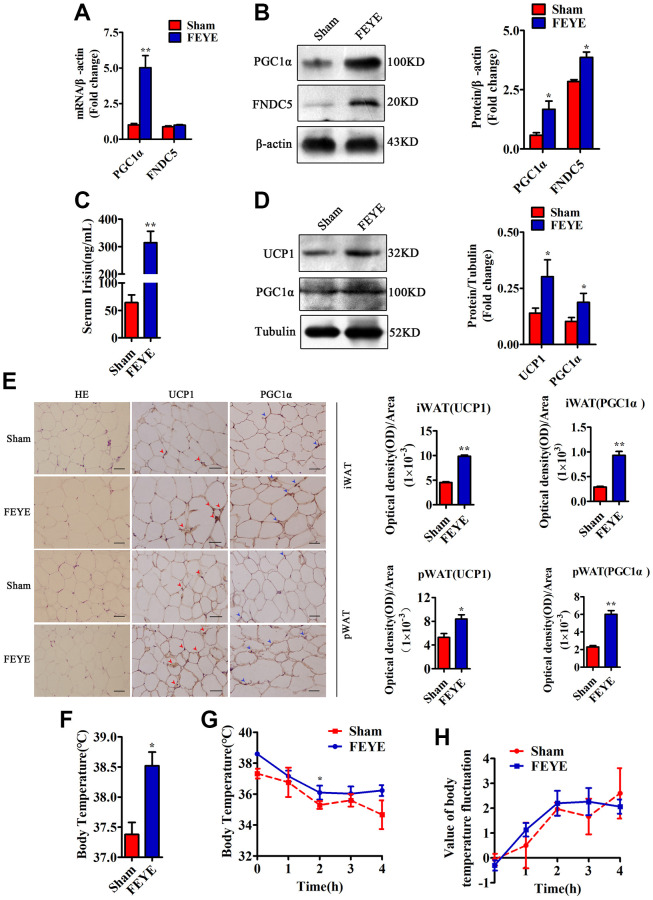
**FEYE builds a bridge between muscle and adipose.** (**A**) FEYE can promote the mRNA expression of PGC1α and FNDC5 in skeletal muscle. Each experiment was performed in triplicate wells; (**B**) Western blot assay suggests that FEYE can promote the protein expression of PGC1α and FNDC5 in skeletal muscle. Each experiment was performed in triplicate wells; (**C**) ELISA assay shows that FEYE can significantly increase the expression of Irisin in serum. Each experiment was performed in triplicate wells; (**D**) Western blot analysis showed FEYE can significantly enhance the expression of UCP1 and PGC1α protein in inguinal adipose tissue (iWAT); (**E**) HE staining of inguinal adipose tissue (iWAT) and perirenal adipose tissue (pWAT) and immunohistochemical staining of UCP1 and PGC1α protein (red arrows indicate the yellow staining sites of UCP1, and blue arrows indicate the yellow staining sites of PGC1α) and analysis of the ratio of optical density to immunostaining area; (**F**–**H**) At room temperature, FEYE can promote heat production in mice (**F**) (*n* = 5/group), Each experiment was performed in quintuplicate wells. At 4°C, the body temperature of mice in the FEYE group is always higher than that in the sham group (**G**) (*n* = 3/group). In addition, in a cold environment, the temperature fluctuation is more stable in different time periods (**H**) (*n* = 3/group). (Bar = 100 μm); ^*^P < 0.05, ^**^P < 0.01.

To explore whether FEYE can build a bridge between muscle and fat, we tested the expression levels of related factors in skeletal muscle, serum and adipose tissue. First, we detected that PGC1α and FNDC5 in the FEYE group were significantly over-expressed from isolated skeletal muscles of mice (Figure 3A, 3B). According to the results of the ELISA assay, the secretion of Irisin significantly increased in FEYE-treated mice serum (Figure 3C). Studies have shown that Irisin plays an important role in the transformation of WAT to BAT [33]. In order to prove whether the increase of Irisin in the serum can cause browning of adipose tissue, we further examined the changes of fat-related genes in WAT. The results showed that FEYE promoted the high expression of UCP1 and PGC1α in WAT (Figure 3D). The results of immunohistochemical staining indicated that FEYE increased the positive expression of UCP1 and PGC1α in iWAT and pWAT (Figure 3E). Therefore, our findings jointly emphasize that FEYE can mediate the browning of WAT by enhancing the expression of PGC1α and FNDC5 in mice.

To confirm the effect of FEYE on heat production, we measured the rectal temperature when the mice were treated at room temperature and 4°C. The basal body temperature of mice in the FEYE group was significantly higher than that in the sham group (Figure 3F). When the mice stimulated with 4°C for 4 h, the FEYE-stimulated mice always maintained a high body temperature. Importantly, although the body temperature of mice in the two groups showed a sharp downward trend from 0 to 2 h, the body temperature showed a significant difference between sham group and FEYE group at 2 h of cold treatment. Otherwise, during the period of 2 to 4 h, the body temperature of the sham group still showed a downward trend, while the body temperature of the FEYE group mice was relatively stable (Figure 3G). The results showed that the temperature fluctuation of mice in the FEYE group was smaller than that in the sham group, and it demonstrated FEYE could enhance the tolerance to cold environment (Figure 3H). Hence, our research results jointly emphasize that FEYE can promote the secretion of Irisin in mice skeletal muscle and mediate WAT browning.

### Component analysis of FEYE

**Figure 4:**
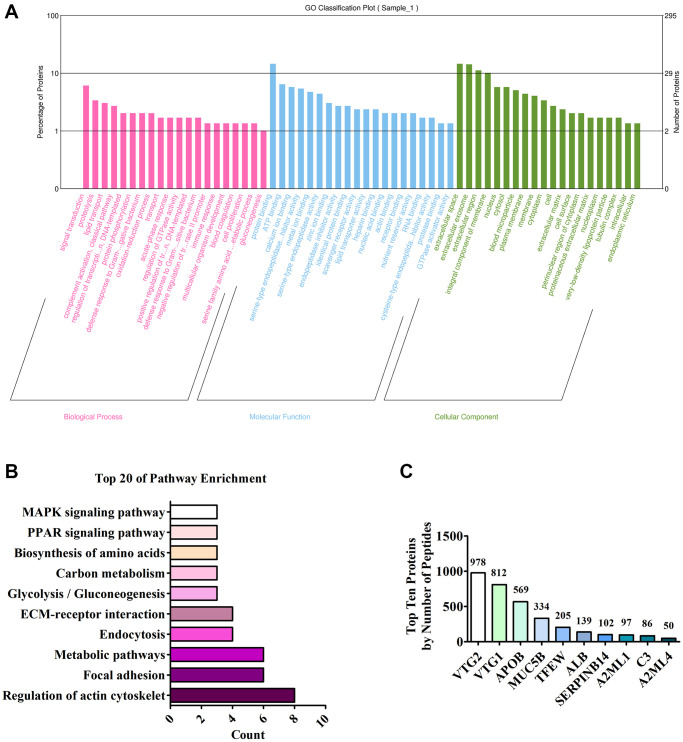
**Analysis of LC MS/MS detection results.** (**A**) Annotation of the GO function of the sample; (**B**) Annotation of the KEGG pathway of the sample; (**C**) Top ten proteins based on the number of peptides.

The full spectrum of FEYE was identified using LC MS/MS method to determine which components in FEYE can promote the growth and development of muscle. A total of 296 proteins were screened. In order to understand the function of these proteins, GO analysis was performed on it, showing that these protein were mainly enriched in signal transduction, protein binding, and extracellular space (Figure 4A). In order to understand the main metabolic pathways and signal transduction pathways involved in proteins, enrichment of KEGG Pathway was carried out. It was found that proteins were the most abundant in signal pathways such as regulation of actin cytoskeleton, focal adhesion, metabolic pathways, and endocytosis (Figure 4B). The top ten proteins were listed according to the number of peptides (Figure 4C). See Supplementary Table 1 for details of the top 10 proteins. After analysis, we found that VTG2 protein is the most abundant component in FEYE. Therefore, we predicted that VTG2 protein played an important role in muscle growth and metabolism.

### *In vitro*, the effect of VTG2 on C2C12 cells and 3T3-L1 cells

**Figure 5:**
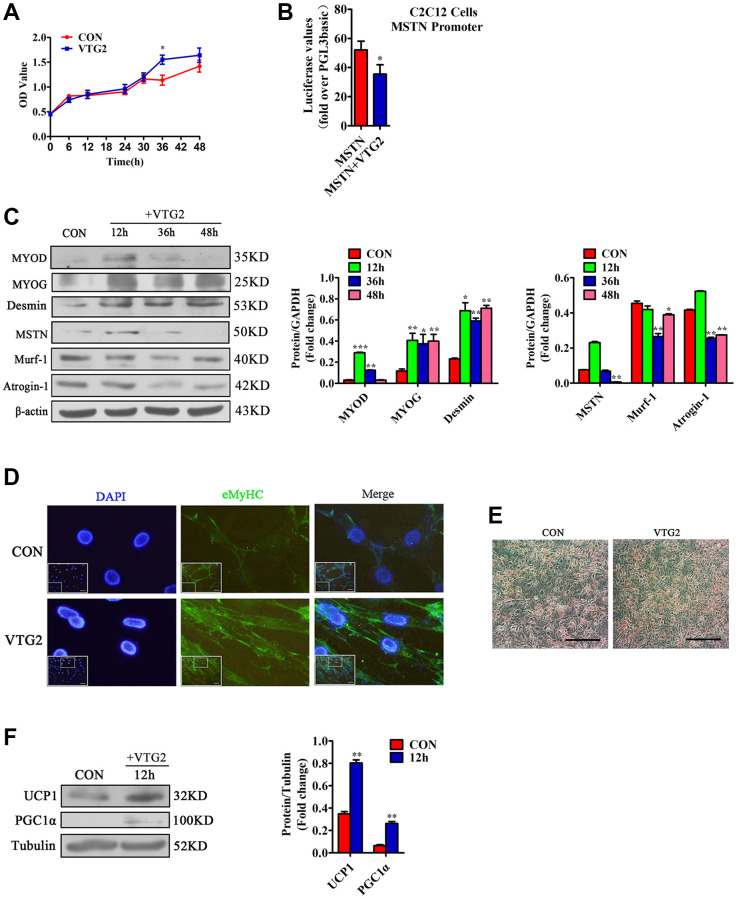
**VTG2 promotes the proliferation of C2C12 cells.** (**A**) CCK-8 assay suggests that when VTG2 (5 ng/mL) is used to treat the cells for 36 h, they show significant proliferation. Each experiment was performed in quadruplicate wells; (**B**) Luciferase method is used in C2C12 cells. VTG2 can inhibit the activity of the MSTN promoter. Each experiment was performed in triplicate wells; (**C**) VTG2 (5 ng/mL) with the same concentration is used to treat C2C12 cells for different times (0 h, 12 h, 36 h, 48 h). Western blot assay suggests that VTG2 can promote the protein expression of MYOD, MYOG, and Desmin in C2C12 cells and inhibit the protein expression of muscle hydrolytic factors MSTN, Murf-1, and Atrogin-1. Each experiment was performed in triplicate wells; (**D**) During the differentiation of C2C12 cells, VTG2 (5 ng/mL) was added and treated for 36 hours. Immunofluorescence staining showed that VTG2 can promote myotube formation, (Bar = 100 μm); (**E**) VTG2 promotes the formation of lipid droplets; (**F**) VTG2 (5 ng/mL) is used to treat 3T3-L1 cells for 12 h, and the Western blot assay suggests that VTG2 can promote the protein expression of UCP1 and PGC1α in 3T3-L1 cells. Each experiment was performed in triplicate wells, (Bar = 200 μm); ^*^P < 0.05, ^**^P < 0.01.

To confirm the effective concentration of VTG2 in C2C12 cells, we diluted it to 0, 0.5, 5, 50, and 500 ng/mL and added it to C2C12 cells at the concentration gradient. We found that when the concentration of VTG2 was 5 ng/ mL, the factors such as MSTN, MYOD and MYOG changed most significantly (Supplementary Figure 2). Therefore, we finally determined that the effective concentration of VTG2 was 5 ng/mL. In order to prove whether VTG2 plays a role *in vitro*, the results of the CCK-8 method showed that compared with the control group, the proliferation rate of the VTG2 group was faster, and it was most obvious at 36 h (Figure 5A). In order to verify the relationship between VTG2 and MSTN, the C2C12 cells were co-transfected with MSTN promoter and VTG2. The result of luciferase assay showed that VTG2 directly inhibited the transcription activity of MSTN (Figure 5B). Later, we found that VTG2 in C2C12 cells further promoted the protein expression of MYOD, MYOG and Desmin, and inhibited the expression of MSTN, Murf-1 and Atrogin-1 (Figure 5C), and the effect was most obvious at 36 h, which is consistent with the CCK-8 assay. In addition, when VTG2 was added to the differentiation medium of C2C12 cells for 36 h, the results showed that VTG2 accelerated the formation of C2C12 cells myotubes (Figure 5D). Hence, when the concentration is 5 ng/mL, VTG2 can directly inhibit the transcription of MSTN and promote the proliferation of C2C12 cells and the formation of myotubes.

In order to prove whether VTG2 acts on 3T3-L1 cells alone and changes, the 3T3-L1 cells were induced and differentiated, and then treated with 5 ng/mL VTG2 for 12 h to prove whether it can affect the metabolism of fat. Our results indicated that VTG2 promoted the formation of lipid droplets, and made brown fat-related factors UCP1 and PGC1α exhibit high expression levels (Figure 5E, 5F).

## DISCUSSION

Many metabolic diseases, including diabetes, chronic kidney disease, and cancer, can cause whole-body muscle atrophy and make people become emaciated [34, 35]. This situation can not only lead to the loss of body muscle mass, but also increase the mortality of humans. At the same time, this can also lead to the decline of animal meat quality in animal husbandry. The growth of muscle mainly depends on changes in the muscle fiber area after birth, and the increase or atrophy of muscle fiber affects the muscle mass [36, 37]. Studies have shown that mice eating eggs significantly increased body weight and reduced fasting blood glucose and insulin [38]. And whole egg intake increases the myofiber protein synthesis reaction after exercise to a greater extent than egg whites intake [39]. Researchers in the food field have found that egg yolk is the main nutrient provider for poultry embryo breeding, and the amounts of various nutrients increase significantly after fertilization [32]. Another study proved that egg yolk extract promotes the proliferation and myogenic differentiation of C2C12 myoblasts [40]. Therefore, we further proved the function of FEYE *in vivo* and *in vitro*. Our findings indicate that FEYE not only increases the area of muscle fibers, but also accelerates energy expenditure by increasing BAT. It was further proved *in vitro* that VTG2 was involved in these results. Our findings reveal that FEYE can promote the increase in muscle mass, fat browning and thermogenesis in mice.

The mechanism of FEYE in promoting muscle mass may be mediated by many factors. First, MSTN is a negative regulatory factor of muscle development. Previous studies proved that inhibition of MSTN can promote muscle development in a variety of ways [9–13]. However, our findings indicated that FEYE effectively inhibited the expression of MSTN in muscle and reduced the secretion of MSTN in the serum. FEYE also significantly inhibited the expression of muscle proteolytic factors Murf-1 and Atrogin-1, and increased the expression of skeletal muscle growth factors MYOD and MYOG. Our study showed that FEYE promoted muscle development by inhibiting the expression of MSTN. Second, muscle development is associated with the AKT signaling pathway in animal bodies. Muscle development is regulated by the PI3K/AKT/mTOR signaling pathway. IGF-1 can transmit the signal to P13K and AKT in turn by the receptor on cell membrane, and AKT transmits the signal to mTOR and FOXO to promote the synthesis of protein and inhibit the degradation of protein, thereby enhancing the growth of muscle fiber [41]. Similarly, our findings showed that FEYE could activate the AKT signaling pathway, thereby promoting the growth and development of muscle. Third, FEYE may promote muscle development by delivering VTG2. The recombinant protein VTG2 directly inhibited the activity of the MSTN promoter and accelerated the proliferation of muscle cells, thereby not only promoted the expression levels of MYOD, MYOG and other factors, but also inhibited the expression levels of MSTN, Atrogin-1 and other factors. This finding is basically consistent with the results of the animal experiments. The present work reveals a new factor called VTG2, which can inhibit MSTN. The results can provide an important scientific basis for preventing muscle atrophy-related diseases and improving animal meat products.

The mechanisms of FEYE affecting on fat metabolism may be to stimulate the expression and Irisin secretion in the muscle and serum. FNDC5 is a membrane binding protein that can be translated and modified to secrete Irisin and can be expressed in myocardial, skeletal, and smooth muscle cells [42, 43]. Related studies have shown that FNDC5 was activated by upstream factor PGC1α in skeletal muscle and secreted Irisin to serum, leading to the browning of WAT [44]. In addition, primary white adipocytes treated with FNDC5 could stimulate the activation of UCP1 and other browning genes. Injection of adenovirus vector expressing Irisin also could cause the browning of subcutaneous adipose tissues in mice [45]. Our results show that FEYE could not only increase the expression of FNDC5 and the activator PGC1α in muscle, but also enhance the secretion of Irisin in the serum, thereby increasing the high expression of brown fat marker UCP1 in mice adipose tissues. Moreover, the reason that FEYE stimulates the browning of WAT may be mediated by VTG2. The recombinant protein VTG2 was added into 3T3-L1 cells after induction and differentiation. It was found that VTG2 promoted the expression level of brown fat marker factors UCP1 and PGC1α. These results suggest that FEYE promotes the browning of white adipose by increasing the expression and secretion of Irisin. The browning reaction may be medicated by VTG2.

In addition to muscle development and fat metabolism, FEYE also has a certain effect on the metabolism of the whole body. First of all, we found that FEYE can increase the weight of mice compared with the sham group, but does not affect the weight of fat. This finding may be due to the increase in the area of muscle fibers in the muscle of mice. Second, FEYE can increase heat production in animals. If the BAT of male donor mice is transplanted into another recipient mice with similar physiological conditions, then the recipient mice has better cold tolerance [46]. The mechanism of heat generation is that BAT is responsible for burning the lipids in white fat, and they release heat through the uncoupling protein UCP1 [47]. Studies have found that the rectal temperature is also significantly increased when browning is mediated by MSTN-knockout mice [48]. The lack of Smad3 even induced the browning reaction of white fat, and the change trend of rectal body temperature measured in different time periods after cold stimulation in mice was almost consistent with this study [49]. Inhibition of ActRIIB increased cold resistance of mice was also caused by the increase of BAT [50]. No matter which factor or condition changes lead to the increase of animal body temperature, it is mediated by the increase of BAT. Therefore, the increase of heat production in the FEYE group may be due to the increase of BAT mediated by FEYE, causing the body to emit more heat. The heat production from BAT plays a vital role in the metabolism of whole body.

**Figure 6:**
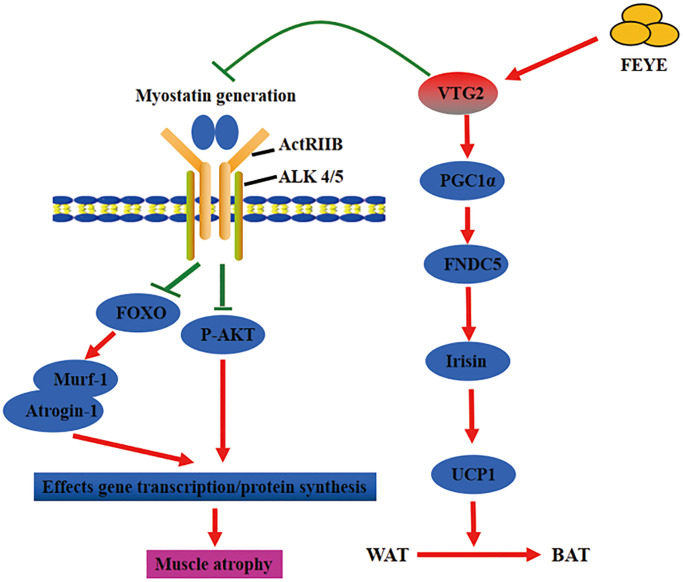
**Fertilized egg yolk extract (FEYE) not only promotes muscle growth and development by delivering VTG2, but also stimulates the browning reaction of white fat.** The green line represents a negative effect, and the red line represents a positive effect. On the one hand, VTG2 inhibits the binding of MSTN and its receptor, thereby promoting P-AKT activity and inhibiting the expression levels of Murf-1 and Atrogin-1. These pathways promote muscle growth. On the other hand, VTG2 can stimulate the expression level of PGC1α, an upstream activator of FNDC5, leading to an increase in Irisin secretion, which can promote the browning of white fat.

In short, FEYE fed to mice not only inhibited muscle atrophy, but also caused browning of WAT. The two different results may be mediated by the same factor (Figure 6). This work enhances our in-depth understanding on the function of FEYE. In addition, some functions of VTG2 were discovered through the study of FEYE. The above results has important scientific significance for improving human muscle atrophy and muscle growth retardation. At the same time, these results play an important role in promoting the development of animal husbandry meat products, and even turning VTG2 into a healthy product for future consumption.

## METHODS

### Preparation and application of FEYE

Fertilized egg yolk was separated from the egg (Shanxi Kangmu Farm, China), and the thin film attached to the yolk surface was peeled off gently. The egg yolk was placed in a clean container and stirred evenly. The yolk was added with PBS buffer at the proportion of 7:3 and continuously stirred to mix. The solution was centrifuged at 3343 g for 20 min. The supernatant was removed, and the precipitate was FEYE, which was used for intragastric administration of experimental mice. The mice were given with FEYE at fixed time every day for a total of 24 d. The initial dose was 0.6 mL, which was increased progressively to 0.8 mL at the maximum, to ensure the adaptation of mice.

### Cold stimulation

The cold tolerance was evaluated by placing the mice in a separate cage, keeping them at 4°C for 4 hours, and measuring the body temperature with a rectal thermometer every hour.

### Experimental animals and sample collection

Thirty healthy wild-type C57BL/6 male mice aged 6 weeks were purchased from Beijing Charles River and placed in the individual ventilated cage system (IVC-system) of the Animal Feeding Center of Shanxi Agricultural University for breeding. The mice were divided into sham group and FEYE group, with 15 mice for each. PBS was given to the sham group by gavage, while the same volume of fertilized egg yolk extract was given to the FEYE group by gavage. In a 12:12 h light/dark cycle, the mice were kept at a constant temperature of 23°C and were given free access to water and food for 24 d. Weight was measured every 2 d. On the 24th day, rectal thermometer was used to measure the normal body temperature and the body temperature after cold violence of mice in the two groups. Then the mice were sacrificed and serum, gastrocnemius (GAS), extensor digitorum longus (EDL), soleus (SOL), inguinal (iWAT), and perinephric (pWAT), were collected in sequence. The removed tissues were weighed, fixed, and frozen. All animal care and experimental protocols complied with the Animal Management Rule of the Ministry of Health, People’s Republic of China (Documentation No. 55, 2001) and the Guide for the Care and Use of Laboratory Animals published by the United States National Institutes of Health (Publication No. 85–23, Revised 1996), and the Global Research Animal Guide. At the same time, it was approved by the Animal Medicine Committee of Shanxi Agricultural University.

### Collection of serum

After the gavage model was established successfully, blood was collected from the retro-orbital vein and was incubated at room temperature (RT) for 30 min. The blood sample was centrifuged at 3000 rpm for 5 min and then at 10000 rpm for 10 min to obtain serum. ELISA kit was used to determine the contents of Irisin (Phoenix Pharmaceuticals, EK-067-29, USA) and MSTN (QIYBO, QIY-01723, China) in the serum of mice.

### Preparation of tissue sections, HE staining, and immunohistochemical staining

The tissues were fixed with 4% paraformaldehyde for 24-48 h. The tissue was dehydrated and transparent with gradient ethanol and xylene, and then immersed in wax for 3 hours before embedding. After drying and fixing into blocks, tissue slices with a thickness of 0.5 μm were prepared. Collect several tissue slices, and use gradient ethanol and xylene to dehydrate and transparent. The tissue slices were stained with hematoxylin and eosin and then sealed with neutral gum. The adipose slices were detected by immunohistochemistry with anti-UCP1 (1:50, Proteintech, 23673-1-AP, Rabbit) and -PGC1α (1:50, Proteintech, 66369-1-1g, Mouse) antibodies. The secondary anti-Mouse (1:150, CWBIO, CW0102S, China) and anti-rabbit (1:150, CWBIO, CW0103S, China) for 1 h at 37°C. After mounting the slides with neutral gum, observe the sections through a confocal microscope (Olympus, FV1000, Japan).

### Cell culture and treatment process

C2C12 cells were purchased from Beijing Zhong Qiao Xin Zhou Biotechnology Co., Ltd., (China). Recombinant protein VTG2 (Mybiosource, MBS1265583, USA) was obtained from Mybiosource Company. The cells were cultured in a high glucose medium (BI, 01-052-1ACS, Israel) containing 10% fetal bovine serum (Sciencell, 0510, USA), and then differentiated with 2% horse serum (Solarbio, S9050, China) when the cells had grown to about 70%. The cell culture was carried out in a constant-temperature incubator containing 5% CO_2_. The use concentration of FEYE is 10 mg/mL, and the use concentration of VTG2 is 5 ng/mL.

3T3-L1 cells were purchased from Beijing Zhong Qiao Xin Zhou Biotechnology Co., Ltd. The cells were cultured in a high glucose medium (BI, 01-052-1ACS, Israel) containing 10% fetal bovine serum (Sciencell, 0510, USA). When the cell growth density reaches about 70%, the Adipogenesis Detection Kit (Abcam, ab133102, USA) is used for differentiation, and the differentiation time is 7 days. VTG2 is added to the differentiation medium for subsequent detection 12 hours before the sample is collected. The entire process of cell culture should be carried out in a constant temperature incubator containing 5% CO_2_.

### Cell transfection and reporter gene assay

In cell transfection, C2C12 cells were inoculated into 24-well plate in advance. When the cell density was about 80%, 1.2 μL of transfection reagent Lipofectamine 2000 (Invitrogen, 11668-019, USA) was mixed with 800 ng of plasmid for transfection. PGL3-basic-MSTN (provided by Prof. Zhang Liping, the restriction sites are *MluI* and *XhoI*) was used as the fluorescent reporter gene [51]. After transfection, the cells are cultured in an incubator for 6 h, and then replaced with the differentiation medium without double antibodies and added with VTG2 protein and cultured for 12 h. The effect of VTG2 on MSTN promoter activity was detected by Luciferase reporter gene detection kit (Promega, E1910, USA).

### Immunofluorescence of cells

After cells attached to the slide, the culture medium was discarded and the cells were washed with PBS buffer three times. The cells were fixed with 4% cold paraformaldehyde for 30 min and washed with PBS buffer three times. Then the cells were added with 0.1% Triton-100 for 10 min to break the cell membrane and washed with PBS buffer three times. Goat serum blocking solution (5%) was added and discard it after blocking for 30 min. The cells were directly added with anti-eMyHC (1:50, Santa Cruz, SC-53091, Mouse) and incubated overnight at 4°C. The cells were washed again with PBS buffer three times. The secondary antibody anti-mouse (1:150, CWBIO, CW0102S, China) was added, and the culture was placed in a constant-temperature incubator at 37°C for 1 h. Then the cells were washed with PBS buffer three times. Sealing with anti-fluorescence attenuation sealing tablets containing DAPI (Solarbio, S2110, China), and taking pictures under a microscope.

### Cell proliferation detected by CCK-8 method

The cell suspension containing 2000 cells was added to a 96-well plate, and the total system was 100 μL. The cells were pre-cultured for 24 h, and then VTG2 was added to the medium, and normal medium was added to the control group. After that, add 10 μL of CCK-8 solution (YEASEN, 40203ES80, China) to each well. After incubating for 2 h, the absorbance at 450 nm was measured by a microplate reader.

### Real-time fluorescence quantitative PCR

Primer 3 Plus was used to design primer sequences and synthesize them online (Supplementary Table 2). The quality of the extracted RNA was detected by 1% agarose gel electrophoresis. cDNA was synthesized according to the instructions of reverse transcription kit (Vazyme, R223-01, China). Mix SYBR (Vazyme, Q711-02, China), DEPC water, cDNA and upstream and downstream primers into a 10 μL system in proportion to perform PCR reaction. All samples were processed in triplicate on real-time Step One software system. Results were calculated based on ΔΔCT value.

### Western blot

BCA method was used to measure the concentration of the extracted protein samples and calculate the loading amount. SDS-PAGE electrophoresis was performed and the gel containing the target protein was transferred to the NC membrane. The membrane was sealed with 5% non-fat milk powder blocking solution for 1 h. The membrane was incubated with the primary antibody overnight at 4°C. Primary antibodies include: anti-MYOD (1:1000, Proteintech, 66214-1-IG, Mouse/1:1000, Abcam, ab16148, Mouse), anti-MYOG (1:500, Bioss, bs-3550R, Rabbit), MSTN (:500, Proteintech, 19142-1-AP, Rabbit), anti-Desmin (1:500, Proteintech, 16520-1-AP, Rabbit), anti-UCP1 (1:1000, Abcam, ab10983, Rabbit), anti-PGC1α (1:800, Proteintech, 66369-1-IG, Mouse), anti-FNDC5 (1:500, Proteintech, 23995-1-AP, Rabbit), anti-Murf-1 (1:1000, SANTA CRUZ, sc-398608, Mouse), anti-Atrogin-1(1:500, Absin, abs136595, Rabbit), anti-phospho-AKT (1:500, Bioss, bs-10996R, Rabbit), anti-AKT (1:500, Bioss, bsm-33278M, Mouse), anti-Tubulin (1:2000, Proteintech, 11224-1-AP, Rabbit) and anti-β-actin (1:2000, CWBIO, CW0096M, Mouse). Subsequently, HRP-conjugated anti-mouse IgG (1:20000, CWBIO, CW0102S) or anti-rabbit IgG (1:20000, CWBIO, CW0156S) were incubated for 1 h after washing with TBST 6 times. According to the manufacturer’s instructions, use ECL (ComWin Biotech Co., Ltd.; catalog number CW0049S) to enhance chemiluminescence detection. Band densities were quantified using Image J. The target protein band density was normalized based on the density of β-actin or Tubulin in the same samples.

### Full-spectrum identification of protein

Dilute the sample and add 10 mM DTT, and the reaction will be reduced at 56°C for 1 h. The sample was added with 55 mM iodoacetamide to perform photophobic reaction at room temperature for 45 min. The sample was then added with 100 μL of 25 mm NH_4_HCO_3_ to clean for 10 min. The sample was dehydrated twice with NH_4_HCO_3_ and 50% acetonitrile solution and dried in vacuum. The sample was then added with 12.5 ng/μl trypsin and incubated at 37°C overnight to facilitate enzymatic hydrolysis. Sixty percent CAN-separated peptide was collected to make C18 ZipTip (Millipore, USA) desalination treatment. About 30 μL of solvent C was added to each sample to prepare suspension, and nano-LC was used to separate. Results were analyzed by online electrospray ionization tandem mass spectrometry. The experiment was carried out on Nano ACQUITY UPLC system (Waters Corporation, USA). A total of 10 μL of the peptide sample was loaded to a trap column (Thermo Scientific, USA; Acclaim PepMap C18, 100 μm × 2 cm) at 10 μL/min. The sample was separated at a 90 min linear gradient in an analytical column (Acclaim PepMap C18, 75 μm × 15 cm). The column was balanced for 10 min under the initial conditions. The flow rate of the column was controlled at 300 nL/min, and the voltage of electrospray was 2 kV. Q-Exactive mass spectrometer was ran in data-dependent acquisition mode and switched automatically between MS and MS/MS acquisition. The full-scan spectrum was obtained at a mass resolution of 70K (m/z 300–1800).

### Data analysis

SPSS software was used to detect difference in gene and protein expression between the control and experimental groups by one-way and two-way ANOVA. Data presented as means ± SD.

### Availability of data and materials

All data generated or analysed during this study are included in this published article and its supplementary information files.

### Ethics approval and consent to participate

All animal operations were carried out in accordance with the “Guidelines for the Care and Use of Laboratory Animals of Shanxi Agricultural University” and were approved by the Animal Medicine Committee of Shanxi Agricultural University.

## Supplementary Materials

Supplementary Figures

Supplementary Tables
