# Supplementary material for: Vitellogenin 2 promotes muscle development and stimulates the browning of white fat
Source: Aging (Albany NY). 2021 Oct 5;13(19):22985–3003. doi: 10.18632/aging.203590 (PMC8544334; doi:10.18632/aging.203590)
Supplement: Supplementary Figures [file aging-13-203590-s001.pdf]

SUPPLEMENTARY FIGURES

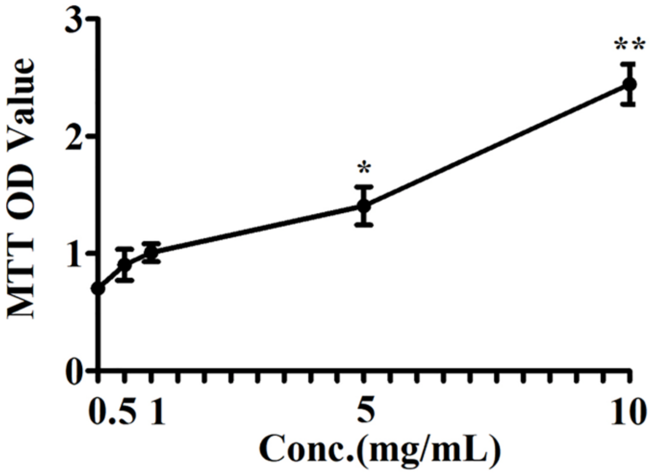

Supplementary Figure 1. The MTT method demonstrated the most effective concentration of FEYE on C2C12 proliferation.

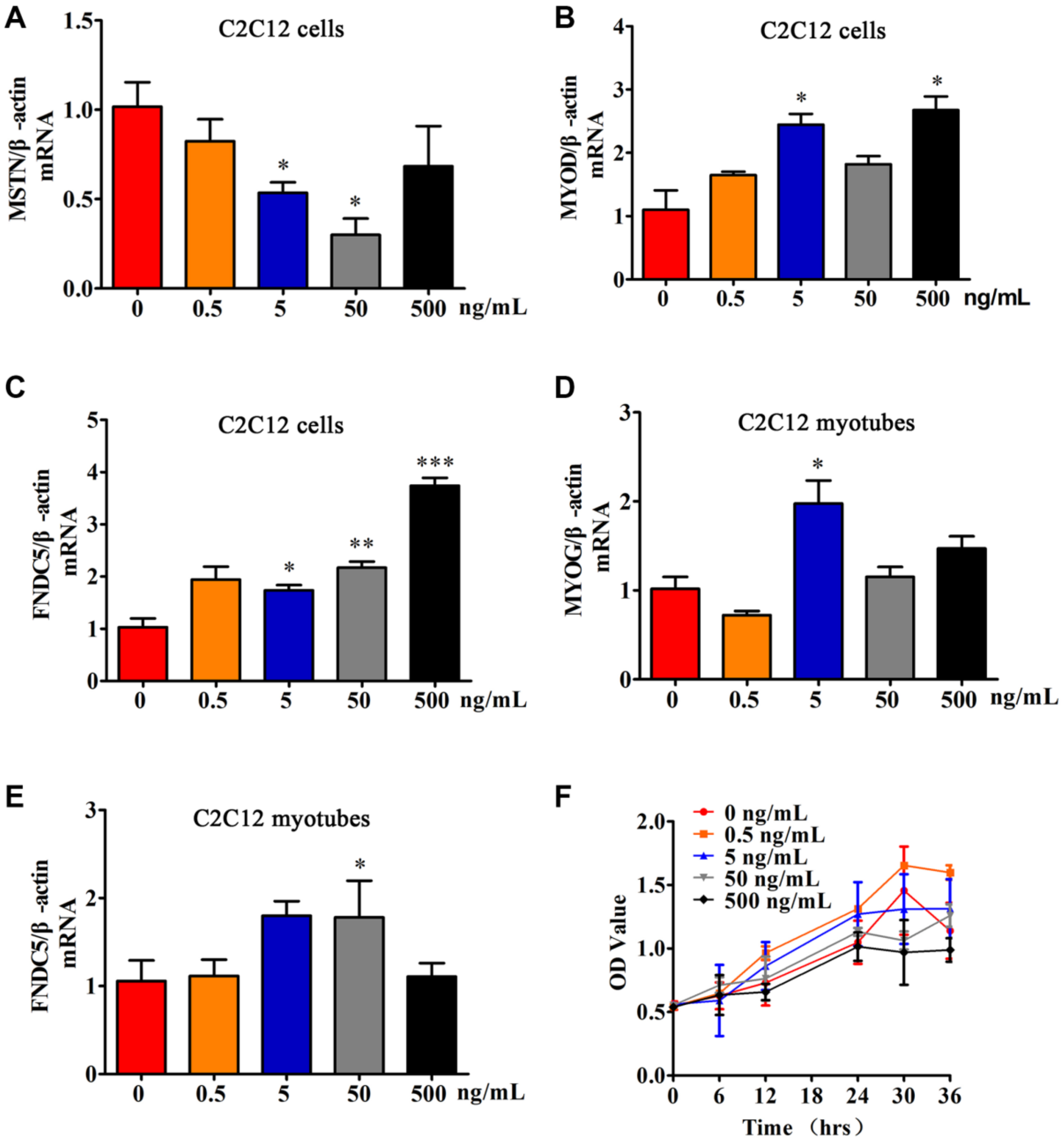

Supplementary Figure 2. VTG2 promotes the proliferation and differentiation of C2C12 cells. (**A**–**C**) VTG2 at different concentration gradients is added during the proliferation of C2C12 cells to act for 36 h. When the concentration of VTG2 is 5 ng/mL, it can not only significantly inhibit the expression of MSTN mRNA (**A**) but can also enhance the mRNA expression of MYOD (**B**) and FNDC5 (**C**); (**D**, **E**) During the differentiation of C2C12, different concentrations of VTG2 were added for 36 hours. When the concentration of VTG2 is 5ng/mL, it can promote the mRNA expression of MYOG (**D**) and FNDC5 (**E**); (**F**) VTG2 at different concentration gradients is used to treat C2C12 cells during the proliferation. CCK-8 assay shows that different concentrations of VTG2 have different influence on the proliferation of cells; $^{*}P < 0.05$, $^{**}P < 0.01$, $^{***}P < 0.001$.
