# Supplementary material for: Vitellogenin 2 promotes muscle development and stimulates the browning of white fat
Source: Aging (Albany NY). 2021 Oct 5;13(19):22985–3003. doi: 10.18632/aging.203590 (PMC8544334; doi:10.18632/aging.203590)
Supplement: Supplementary Tables [file aging-13-203590-s002.pdf]

SUPPLEMENTARY TABLES

Supplementary Table 1. List of top ten protein details in FEYE components.

UniProt Entry	Gene name	Blast Description Annotation	Peptides	KEGG
F1NFL6	VTG2	vitellogenin-2-like	978	gga:424533
P87498	VTG1	vitellogenin-1-like	812	gga:424547
F1NV02	APOB	apolipo B-100	569	gga:396535
Q98UI9	MUC5B	mucin-5B	334	gga:395381
P02789	TFEW	ovalbumin	205	gga:396241
A0A1D5NW68	ALB	serum albumin	139	gga:396197
P01012	SERPINB14	ovalbumin	102	gga:396058
A0A1D5P2X2	A2ML1	alpha-2-macroglobulin 1	97	gga:418254
A0A1D5P9F9	C3;C3d	complement C3	86	gga:396370
F1NK40	A2ML4	alpha-2-macroglobulin 1	50	gga:100858010

Supplementary Table 2. The sequences of primers.

Primer Name	Primer Sequence 5′→3′	Temperature/°C
MSTN	F: CAGACCCGTCAAGACTCCTAC<br>R: CTGCCAAATACCAGTGCCT	55
MyoD	F: GAATGGCTACGACACCGCCTACTAC<br>R: ACGGGGTCTGGGTTCCCTGTT	55
MuRF-1	F: TGATTCCTGATGGAAACGCT<br>R: TCATTGGTGT TCTTCTTTAC CCTC	60
Atrogin-1	F: TAGCATCGGTATGACTAAGT<br>R: AGTCATATGGCAAGCATAC	55
PGC1-α	F: AGTAAGCACACGTTTATTCACGG<br>R: GTCGCCCTTGTTCGTTCTGT	58
FNDC5	F: TAGGCTGCGTCTGCTTCG<br>R: TGTCCTCCTCCAGGTCCCA	60
MYOG	F: AATGCACTGGAGTTCGGTCC<br>R: AGTTGGGCATGGTTTCGTCT	60
β-actin	F: TTGCTGACAGGATGCAGAAG<br>R: ACATCTGCTGGAAGGTGGAC	60
